# TRIM21 suppresses uterine corpus endometrial carcinoma progression by promoting K48 -linked ubiquitination and degradation of SOAT1

**DOI:** 10.3389/fimmu.2026.1863307

**Published:** 2026-08-17

**Authors:** Hongwei Chen, Di Cui

**Affiliations:** Fuyang Medical College, Fuyang Normal University, Fuyang, Anhui, China

**Keywords:** apoptosis, lipid metabolism, SOAT1, TRIM21, ubiquitination, uterine corpus endometrial carcinoma

## Abstract

**Background:**

Uterine corpus endometrial carcinoma (UCEC) is one of the most common gynecological malignancies, and its progression is closely associated with dysregulated protein homeostasis and metabolic reprogramming. TRIM21 is an E3 ubiquitin ligase implicated in tumor regulation, but its role in UCEC remains unclear.

**Methods:**

Datasets from TCGA, CPTAC, and GEO were used to assess the expression pattern, clinical significance, and cellular distribution of TRIM21 in UCEC. TRIM21 expression was validated by immunohistochemistry, qRT-PCR, western blotting, and immunofluorescence. Gain-of-function assays, including CCK-8, colony formation, Transwell migration, apoptosis assays, lipid droplet staining, and xenograft experiments, were performed to determine the biological effects of TRIM21. Quantitative proteomics, immunoprecipitation coupled with mass spectrometry, co-immunoprecipitation, cycloheximide chase, ubiquitination assays, and rescue experiments were conducted to investigate the underlying mechanism.

**Results:**

TRIM21 was significantly downregulated in UCEC tissues and cell lines and was associated with favorable prognosis and less aggressive clinicopathological features. TRIM21 overexpression inhibited proliferation, migration, lipid droplet accumulation, and tumor growth, while promoting apoptosis. Mechanistically, SOAT1 was identified as a novel interacting protein of TRIM21. TRIM21 negatively regulated SOAT1 by promoting its K48-linked ubiquitination and proteasomal degradation, thereby reducing SOAT1 protein stability. Restoration of SOAT1 partially reversed the inhibitory effects of TRIM21 on malignant phenotypes and its pro-apoptotic effects.

**Conclusion:**

TRIM21 functions as a tumor suppressor in UCEC by promoting K48-linked ubiquitination and degradation of SOAT1. These findings reveal a novel mechanism linking protein homeostasis to lipid metabolic regulation in UCEC and suggest that TRIM21 and SOAT1 may serve as potential biomarkers and therapeutic targets.

## Introduction

Uterine corpus endometrial carcinoma, UCEC, is one of the most common malignancies of the female reproductive system, and its incidence has continued to rise in recent years (1, 2). According to the latest cancer data released by the United States, there were 67880 new cases of endometrial cancer and 13250 new deaths in 2024 (3). Although many patients are diagnosed at an early stage and achieve relatively favorable outcomes after surgery-based treatment, a considerable proportion still develop recurrence, metastasis, or disease progression, resulting in poor prognosis (4–6). Sustained proliferative capacity, enhanced migratory and invasive behavior, and resistance to programmed cell death are recognized as major biological features driving UCEC progression (7, 8). Therefore, identification of key molecules involved in these processes and clarification of their regulatory mechanisms remain of substantial importance for improving diagnosis and treatment.

The ubiquitin proteasome system is a major pathway responsible for maintaining protein homeostasis in eukaryotic cells (9). Through the coordinated actions of E1 activating enzymes, E2 conjugating enzymes, and E3 ubiquitin ligases, substrate proteins undergo ubiquitination, which determines their degradation, subcellular localization, or functional state (10, 11). Among these components, E3 ubiquitin ligases are of particular importance because they confer substrate specificity and thereby play central roles in tumor-related signaling pathways (12, 13). The tripartite motif family comprises a group of RING domain-containing E3 ubiquitin ligases that participate in cell proliferation, differentiation, inflammation, innate immunity, and tumor progression (14, 15). TRIM21 is an important member of this family and was initially recognized for its roles in antiviral defense and autoimmune diseases (16, 17). More recently, increasing evidence has shown that TRIM21 is involved in multiple cancer-related processes, including tumor growth, metastasis, metabolic reprogramming, immune regulation, and resistance to cell death (18–20). In several tumor types, TRIM21 exerts tumor suppressive effects by promoting the ubiquitination and degradation of oncogenic proteins. For example, TRIM21 has been reported to facilitate the degradation of mutant p53, PRMT1, and SIX2, thereby suppressing malignant progression, stemness maintenance, and metastasis (21–23). TRIM21 has also been shown to regulate signaling pathways associated with metastasis and treatment response through substrate-specific ubiquitination (24). In contrast, TRIM21 may also promote tumor progression in certain settings by targeting proteins involved in metabolic adaptation or cell death regulation (25, 26). These findings suggest that the biological role of TRIM21 depends on the nature of its substrates, the type of ubiquitin linkage involved, and the molecular background of specific tumors. However, the expression pattern, biological function, and molecular mechanism of TRIM21 in UCEC remain unclear.

Metabolic reprogramming has emerged as a hallmark of cancer, and abnormal lipid metabolism has attracted increasing attention in recent years (27). Cholesterol metabolism is not only essential for membrane biosynthesis but also contributes to membrane signaling, oxidative stress responses, and cell survival (28, 29). Sterol O-acyltransferase 1(SOAT1) is a key cholesterol-esterifying enzyme that catalyzes the conversion of free cholesterol into cholesteryl esters and promotes their storage in lipid droplets (30). Aberrant SOAT1 expression has been reported in multiple cancers, where it contributes to lipid accumulation, enhanced proliferation and migration, and increased tolerance to metabolic stress (31–33). However, the regulatory mechanism of SOAT1 in UCEC has not been fully defined, particularly with regard to whether its protein stability is controlled by a specific E3 ubiquitin ligase.

In the present study, the expression, biological function, and molecular mechanism of TRIM21 in UCEC were systematically investigated. By integrating public datasets, bioinformatic analyses, *in vitro* and *in vivo* functional experiments, quantitative proteomics, and protein interaction assays, the clinical significance and tumor suppressive role of TRIM21 in UCEC were characterized. Particular attention was given to the regulation of SOAT1 protein stability by TRIM21. These findings provide evidence that abnormal protein homeostasis is linked to lipid metabolic reprogramming in UCEC and may offer a theoretical basis for the development of targeted therapeutic strategies.

## Materials and methods

### Data collection and bioinformatic analyses

Transcriptomic expression data and corresponding clinical information for TRIM21 were obtained from The Cancer Genome Atlas and used for pan cancer expression profiling, differential expression analysis in UCEC, survival analysis, clinicopathological correlation analysis, and stemness analysis. Protein expression data from the Clinical Proteomic Tumor Analysis Consortium were used to validate TRIM21 expression in UCEC. The GSE139555 dataset from the Gene Expression Omnibus was used for single cell transcriptomic analysis. All datasets were normalized before downstream analyses. Pan cancer analysis was performed to compare TRIM21 expression between tumor tissues and matched normal tissues across different cancer types. In UCEC, both unpaired and paired analyses were conducted to assess differential expression, and receiver operating characteristic analysis was used to evaluate the diagnostic value of TRIM21. Kaplan Meier analysis with log rank testing was used to evaluate overall survival and progression free interval. Single cell transcriptomic analysis was performed after quality control, normalization, dimensionality reduction, and clustering, followed by visualization with uniform manifold approximation and projection and cell type annotation based on canonical markers. Stemness analysis was performed by extracting RNAss, DNAss, DMPss, and ENHss scores and assessing their correlations with TRIM21 expression using Spearman analysis.

### Tissue microarray and immunohistochemistry

A UCEC tissue microarray containing 15 tumor tissues and 15 matched adjacent normal tissues was purchased from Shanghai Outdo Biotech Co. Ltd. The research plan has been approved by the Ethics Committee of Shanghai Outdo Biotech Co. Ltd [Approval Number: SHYJS-CP-240201-02(XZ)]. Paraffin-embedded tissue sections were deparaffinized in xylene, rehydrated through graded ethanol, and subjected to antigen retrieval in citrate buffer. Endogenous peroxidase activity was blocked with three percent hydrogen peroxide, and nonspecific binding was blocked with five percent bovine serum albumin. Sections were incubated with a primary antibody against TRIM21 at 4°C overnight, followed by incubation with the appropriate horseradish peroxidase conjugated secondary antibody. Signal detection was performed using diaminobenzidine, and nuclei were counterstained with hematoxylin. TRIM21 expression was semiquantitatively evaluated using the H score method based on staining intensity and the percentage of positive cells. All slides were independently reviewed by two pathologists blinded to the clinical information.

### Cell culture and gene manipulation

Human endometrial cancer cell lines KLE, Ishikawa, HEC-1-B, and AN3CA, together with the normal endometrial epithelial cell (hEEC), were obtained from Pricella Life Technology Co., Ltd and cultured in medium supplemented with 10% fetal bovine serum at 37°C in a humidified incubator containing 5% CO_2_. Stable TRIM21-overexpressing and TRIM21-knockdown cell lines were generated using lentiviral transduction. Briefly, the full-length coding sequence of human TRIM21 was cloned into a lentiviral overexpression vector, and two independent shRNA sequences targeting TRIM21 were cloned into a lentiviral shRNA vector. The target sequences were as follows: shRNA-1, 5′-TGGAAGTGGAAATTGCAATAA-3′; and shRAN-2, 5′-CAATCCGTGGCTGATACTTTC-3′. The corresponding empty vector or non-targeting shRNA vector was used as the negative control. Lentiviral particles for TRIM21 overexpression, TRIM21 knockdown, and the corresponding control vectors were purchased from Tsingke Biotechnology Co., Ltd. AN3CA and HEC-1-B cells were seeded in six-well plates and infected with the indicated lentiviruses according to the manufacturer’s instructions in the presence of polybrene. After 24 h of infection, the culture medium was replaced with fresh complete medium. Stable cells were selected with puromycin for 14 days, and surviving cells were maintained in puromycin-containing medium. The efficiency of TRIM21 overexpression or knockdown was confirmed by quantitative real-time PCR and western blotting before subsequent experiments. For mechanistic studies, cells were transfected with the indicated plasmids or short hairpin constructs, including Flag tagged TRIM21, Myc tagged SOAT1, HA tagged ubiquitin, and the corresponding control vectors.

### RNA extraction and quantitative real time polymerase chain reaction

Total RNA was isolated using TRIzol reagent (Invitrogen, USA), and complementary DNA was synthesized using a reverse transcription kit (Servicebio, Wuhan, China) according to the manufacturer’s instructions. Quantitative real time polymerase chain reaction was performed using a SYBR Green based system (Servicebio, Wuhan, China), with GAPDH serving as the internal control. Relative expression levels were determined via the 2^−ΔΔCt calculation method. All reactions were performed in triplicate to ensure reproducibility. The primer sequences were: CD206, forward 5′- TCCGGGTGCTGTTCTCCTA -3′ and reverse 5′- CCAGTCTGTTTTTGATGGCACT -3′; IL10, forward 5′- GACTTTAAGGGTTACCTGGGTTG -3′ and reverse 5′- TCACATGCGCCTTGATGTCTG -3′; TRIM21, forward 5′- TCAGCAGCACGCTTGACAAT -3′ and reverse 5′- GGCCACACTCGATGCTCAC -3′; GAPDH, forward 5′- GGAGCGAGATCCCTCCAAAAT -3′ and reverse 5′- GGCTGTTGTCATACTTCTCATGG -3′.

### Western blot analysis

Total cellular protein was extracted using lysis buffer supplemented with protease inhibitors and quantified with a bicinchoninic acid assay. Equal amounts of protein were separated by sodium dodecyl sulfate polyacrylamide gel electrophoresis and transferred onto polyvinylidene fluoride membranes. After blocking in five percent nonfat milk, membranes were incubated with primary antibodies against the indicated proteins, followed by species appropriate secondary antibodies. Primary antibodies used in this study were purchased from Proteintech Group (Wuhan, China), including antibodies against TRIM21 (Cat. No.12108-1-AP, 1:1000), SOAT1 (Cat. No. 13202-1-AP), BCL2 (Cat. No. 12789-1-AP, 1:1000), Caspase-3 (Cat. No.19677-1-AP, 1:1000), Cleaved Caspase-3 (Cat. No.25128-1-AP, 1:1000), MYC-tag (Cat. No. 60003-2-Ig, 1:1000), HA-tag (Cat. 66006-2-Ig, 1:1000), Flag-tag (Cat. No. 66008-4-Ig, 1:1000), ubiquitin (Cat. 10201-2-AP and 80992-1-RR, 1:1000) and GAPDH (Cat. No.60004-1-Ig, 1:2000). Protein bands were visualized using enhanced chemiluminescence (Servicebio, Wuhan, China) and quantified by densitometric analysis.

### Immunofluorescence staining and lipid droplet staining

Cells were fixed with 4% paraformaldehyde, permeabilized with 0.1% Triton X-100, and blocked with 5% bovine serum albumin. The cells were then incubated overnight at 4 °C with primary antibodies against TRIM21(67136-1-Ig, Proteintech) and SOAT1(13202-1-AP, Proteintech), followed by fluorescence-conjugated secondary antibodies for 1h at room temperature. Nuclei were counterstained with DAPI. Images were captured using Leica TCS SP8 laser scanning confocal microscope (Leica Microsystems, Wetzlar, Germany). For lipid droplet staining, cells were incubated with Nile Red working solution (C2051S, Beyotime, China), counterstained with DAPI, and analyzed by fluorescence microscopy. Mean fluorescence intensity was quantified to assess intracellular lipid accumulation.

### Measurement of intracellular cholesterol and cholesteryl ester levels

Intracellular cholesterol and cholesteryl ester (CE) levels were measured using a Cholesterol/Cholesteryl Ester Quantification Assay Kit (S0211S, Beyotime Biotechnology, China) according to the manufacturer’s instructions. Free cholesterol and total cholesterol were determined separately, and CE content was calculated by subtracting free cholesterol from total cholesterol. The results were normalized to total protein concentration and expressed relative to the control group. All experiments were performed in triplicate.

### Functional assays *in vitro*

Cell proliferation was evaluated using Cell Counting Kit 8 assays. Cells were seeded into 96 well plates, and OD values at 450 nm were measured at 12 h, 24 h, 48 h and 72 h after incubation with the reagent. Colony formation assays were performed by seeding cells at low density in 6 well plates and culturing them for ten to fourteen days before fixation, crystal violet staining, and colony counting. Cell migration was assessed using Transwell assays. Cells were seeded into the upper chamber, allowed to migrate for the indicated duration, and then fixed, stained, and counted.

### Xenograft assay *in vivo*

For *in vivo* tumorigenesis assays, all animal experiments were reviewed and approved by the Institutional Animal Care and Use Committee of Fuyang Normal University. Female immunodeficient nude mice (BALB/c nude mice, 7 weeks old) were housed under specific pathogen-free conditions with free access to food and water. AN3CA or HEC-1-B cells with stable TRIM21 overexpression or the corresponding control cells were harvested during the logarithmic growth phase, washed with sterile PBS, and resuspended in a mixture of PBS and Matrigel. A total of 5 × 10^6^ cells in 100 μL of suspension were subcutaneously injected into the flank of each mouse. Tumor length and width were measured regularly using calipers, and tumor volume was calculated as follows: volume = 1/2 × length × width². Mice were monitored for tumor growth, body weight, general condition, food and water intake, and signs of distress. The predefined humane endpoints included tumor volume exceeding 1,500 mm³ or the maximum size permitted by the animal ethics committee, tumor ulceration, necrosis or bleeding, impaired mobility, poor feeding and drinking. At the experimental endpoint, mice were euthanized according to institutional animal welfare guidelines. Xenograft tumors were excised, photographed, weighed, fixed in formalin, paraffin-embedded, and sectioned for immunohistochemical staining of TRIM21 (12108-1-AP, Proteintech) and Ki-67 (27309-1-AP, Proteintech).

### Quantitative proteomic analysis and pathway enrichment

For quantitative proteomic analysis, HEC-1-B cells with stable TRIM21 overexpression and the corresponding control cells were prepared in three biological replicates per group. Total protein was extracted using lysis buffer containing 8 M urea, 1 mM PMSF, and 2 mM EDTA. After sonication on ice, the lysates were centrifuged at 15,000g for 10 min at 4 °C, and protein concentrations were determined using a BCA protein assay. For digestion, 100 μg of protein from each sample was reduced with 5 mM DTT at 37 °C for 45 min and alkylated with 11 mM iodoacetamide for 15 min in the dark. Samples were then diluted with 25 mM ammonium bicarbonate, digested overnight at 37 °C with trypsin (Promega, V5280), acidified to pH 2–3 with TFA, and desalted using C18 columns. DIA-based LC-MS/MS analysis was performed using a Vanquish Neo UHPLC system coupled to an Orbitrap Astral mass spectrometer (Thermo Scientific). Peptides were separated on an Easy-Spray PepMap Neo UHPLC column using mobile phase A, 0.1% formic acid in water, and mobile phase B, 0.1% formic acid in acetonitrile. The column temperature was maintained at 55 °C, and 200 ng peptides were loaded for each run. MS1 scans were acquired over a range of 380–980 m/z at a resolution of 240,000. MS2 data were acquired in DIA mode with 299 isolation windows, an isolation window of 2 Th, and HCD collision energy of 25%. Protein abundance values were normalized before differential expression analysis. Differentially expressed proteins between the TRIM21-overexpression and control groups were identified using the thresholds of fold change > 1.5 or < 0.67 and *P* < 0.05. Multiple-testing correction was performed using the Benjamini–Hochberg method, and adjusted *P* values or false discovery rate values were provided where applicable. Heatmaps, volcano plots, and KEGG enrichment analysis were performed. The complete protein expression matrix and differential protein analysis results are provided in **Supplementary Table 1** and **S2**.

### Apoptosis and ultrastructural analyses

Apoptosis was analyzed using Annexin V fluorescein isothiocyanate and propidium iodide double staining (C1062S, Beyotime, China) followed by flow cytometry. Cells were collected, stained according to the manufacturer’s instructions, and analyzed to determine the proportions of early and late apoptotic cells. For ultrastructural analysis, cells were fixed in 2.5% glutaraldehyde, dehydrated through graded ethanol, embedded in resin, sectioned ultrathin, and stained with uranyl acetate and lead citrate before examination under a transmission electron microscope.

### Protein interaction and ubiquitination assays

Immunoprecipitation was performed to enrich TRIM21 and its interacting proteins. Cell lysates were clarified by centrifugation and incubated with a TRIM21 antibody (Cat. No. 83530-3-RR) together with Protein A + G beads (P2108, Beyotime, China). Immunoglobulin G was used as the negative control. The eluted proteins were separated by electrophoresis, visualized by silver staining, and subjected to mass spectrometry to identify candidate TRIM21-interacting proteins. All identified candidate interacting proteins are listed in **Supplementary Table 3**. Co immunoprecipitation experiments were subsequently performed using TRIM21(Cat. No. 83530-3-RR) or SOAT1(Cat. No. 13202-1-AP) antibodies to validate endogenous protein interactions. Molecular docking analysis was conducted using publicly available protein structural data to predict the interaction interface between TRIM21 and SOAT1. To assess the effect of TRIM21 on SOAT1 protein stability, cells were transfected with Flag tagged TRIM21 expression plasmids and treated with the proteasome inhibitor MG132 for 12 h where indicated. Cycloheximide chase assays were performed by adding cycloheximide and collecting cells at 0 h, 4 h, 8 h, and 12 h to evaluate SOAT1 degradation kinetics. For ubiquitination assays, cells were subjected to TRIM21 knockdown or overexpression and cotransfected with Myc tagged SOAT1 and HA tagged ubiquitin plasmids. MG132 was added before harvesting to prevent rapid degradation of ubiquitinated SOAT1. To determine the linkage type, wild type, K48R, and K63R ubiquitin constructs were used, followed by immunoprecipitation of SOAT1 and immunoblotting for ubiquitin. Full-length Flag-tagged TRIM21 and a RING domain-deleted TRIM21 mutant (Flag-TRIM21-ΔRING) were cotransfected with Myc-tagged SOAT1 and HA-tagged ubiquitin. Following MG132 treatment, SOAT1 was immunoprecipitated, and its ubiquitination level was analyzed by immunoblotting.

### Conditioned-medium-induced macrophage polarization

THP-1 cells were treated with phorbol 12-myristate 13-acetate (PMA) for 24 h to induce macrophage differentiation. Conditioned media were collected from AN3CA cells with TRIM21 knockdown or overexpression and their corresponding control cells. After centrifugation to remove cellular debris, the conditioned media were applied to differentiated THP-1 cells. Macrophage polarization was subsequently evaluated by flow cytometric analysis of CD206-positive cells and quantitative real-time PCR measurement of CD206 and IL-10 expression.

### Statistical analysis

All statistical analyses were performed using R software and other appropriate bioinformatic platforms. Group comparisons were conducted using the Wilcoxon rank sum test or paired Wilcoxon signed rank test as appropriate. Survival differences were assessed using the log rank test. Correlation analyses were performed using Spearman correlation coefficients. *P* < 0.05 was considered statistically significant.

## Results

### TRIM21 is downregulated in UCEC and is associated with favorable clinicopathological features and prognosis

**Figure 1:**
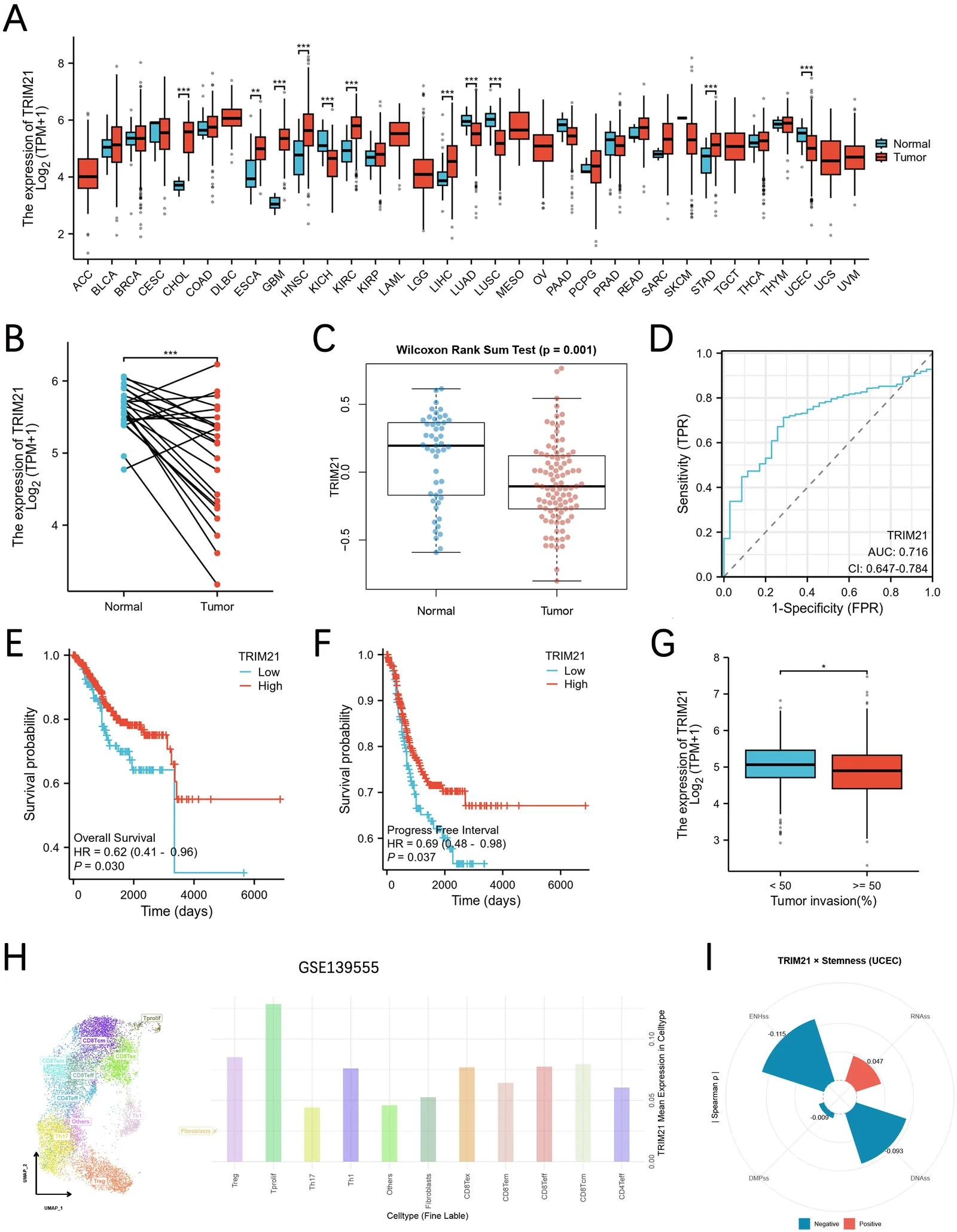
TRIM21 is downregulated in UCEC and is associated with favorable clinical features and prognosis. **(A)** Pan-cancer analysis of TRIM21 expression across TCGA datasets. **(B)** Paired analysis of TRIM21 mRNA expression in UCEC tissues and matched normal tissues from TCGA. **(C)** Validation of TRIM21 protein expression in UCEC using the CPTAC cohort. **(D)** Receiver operating characteristic curve evaluating the diagnostic performance of TRIM21 in distinguishing UCEC tissues from normal tissues. **(E, F)** Kaplan–Meier survival curves showing the association of TRIM21 expression with overall survival and progression-free interval in patients with UCEC. **(G)** Association between TRIM21 expression and tumor invasion in UCEC. **(H)** Single-cell RNA sequencing analysis of TRIM21 expression in different cell populations from the GSE139555 dataset. Left, UMAP plot showing cell clustering and annotation; right, mean TRIM21 expression across different cell types. **(I)** Correlation between TRIM21 expression and stemness indices in UCEC. Data are presented as median with interquartile range or mean ± SD, as appropriate. *P < 0.05, **P < 0.01, ***P < 0.001.

We first examined the expression pattern of TRIM21 across pan cancer datasets from TCGA. TRIM21 showed marked tumor heterogeneity and was decreased in multiple solid tumors (**Figure 1A**). In UCEC, TRIM21 expression was significantly lower in tumor tissues than in normal tissues, and this downregulation was also confirmed in paired samples (**Figure 1B**). Analysis of the CPTAC cohort further demonstrated that TRIM21 protein expression was significantly reduced in UCEC tissues (**Figure 1C**). Receiver operating characteristic analysis showed that TRIM21 had moderate diagnostic value for distinguishing UCEC from normal tissues (**Figure 1D**). Survival analysis indicated that patients with high TRIM21 expression had significantly better overall survival and progression free interval than those with low TRIM21 expression (**Figures 1E, F**). Multivariate regression analysis demonstrated that TRIM21 was an independent prognostic factor in patients with UCEC (**Supplementary Figure 1A**). In addition, TRIM21 expression was significantly decreased in patients with tumor invasion of at least 50%, suggesting an association between low TRIM21 expression and enhanced invasiveness (**Figure 1G**). Single cell transcriptomic analysis further revealed heterogeneous distribution of TRIM21 within the UCEC tumor microenvironment, with relatively high expression in multiple T cell subsets and fibroblasts, particularly in Tprolif cells (**Figure 1H**). Stemness analysis showed that TRIM21 was only weakly correlated with UCEC stemness indices, displaying a very weak positive correlation with RNAss and weak negative correlations with DNAss and ENHss, suggesting that TRIM21 may be associated with reduced epigenetic stemness (**Figure 1I**).

### TRIM21 expression is decreased in UCEC tissues and cell lines

**Figure 2:**
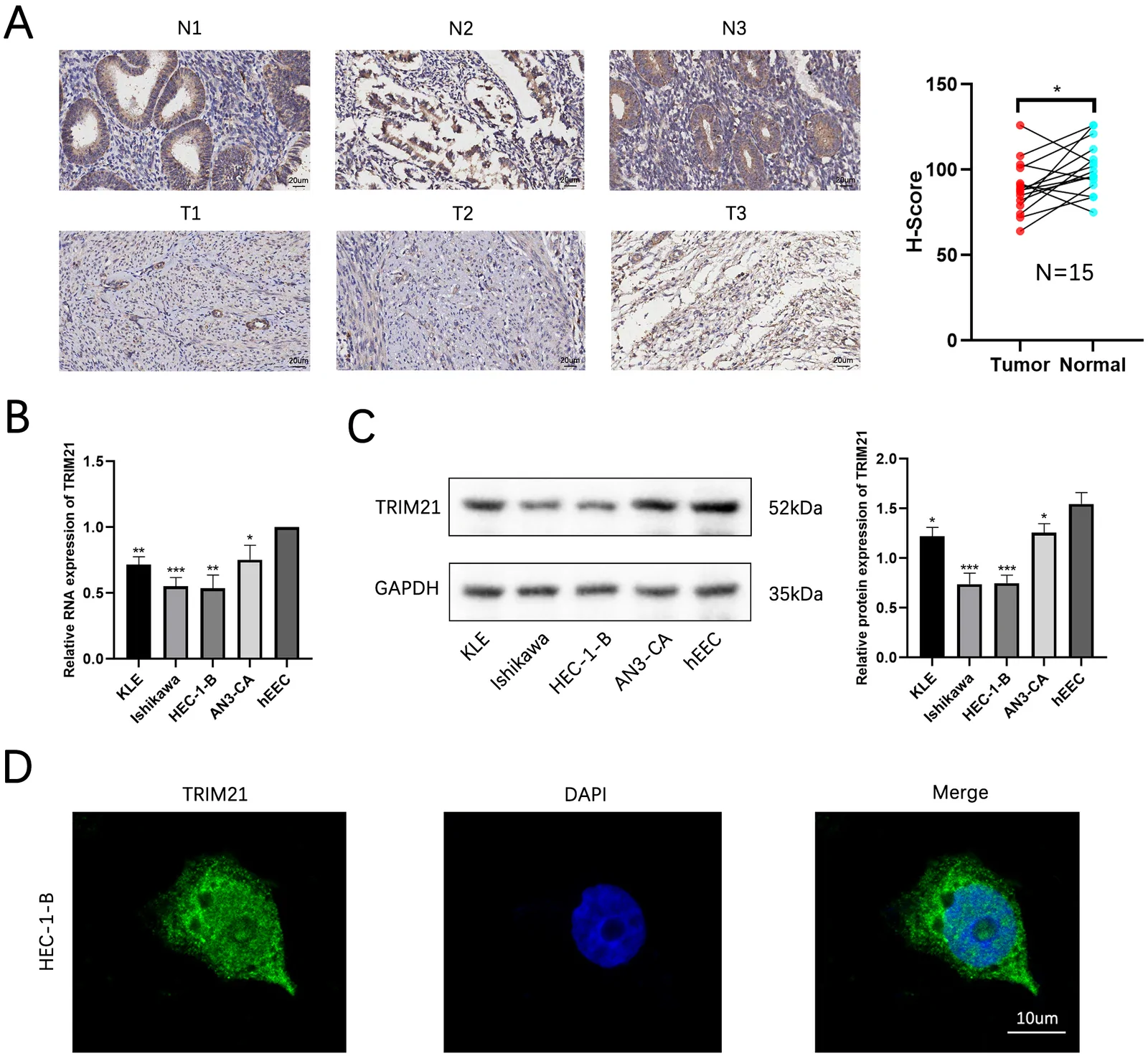
TRIM21 expression is reduced in UCEC tissues and cell lines. **(A)** Representative immunohistochemical staining images of TRIM21 in normal endometrial tissues and UCEC tissues from the tissue microarray, together with quantitative analysis of H-scores. **(B)** Relative mRNA expression of TRIM21 in UCEC cell lines and normal human endometrial epithelial cells, as determined by qRT-PCR. **(C)** Western blot analysis and quantification of TRIM21 protein expression in UCEC cell lines and normal human endometrial epithelial cells. **(D)** Immunofluorescence staining showing the subcellular localization of TRIM21 in HEC-1-B cells. Scale bar, 10 μm. Data are shown as mean ± SD. *P < 0.05, **P < 0.01, ***P < 0.001.

Immunohistochemical staining of the tissue microarray showed that TRIM21 staining intensity was markedly lower in UCEC tissues than in normal endometrial tissues (**Figure 2A**). At the cellular level, quantitative real time polymerase chain reaction demonstrated that TRIM21 messenger RNA expression was significantly reduced in multiple UCEC cell lines, including KLE, Ishikawa, HEC-1-B, and AN3CA, compared with normal human endometrial epithelial cells (**Figure 2B**). Western blot analysis further confirmed that TRIM21 protein levels were also decreased in UCEC cell lines, consistent with the transcriptional findings (**Figure 2C**). Immunofluorescence staining showed that TRIM21 was distributed in both the cytoplasm and nucleus (**Figure 2D**).

### TRIM21 suppresses proliferation, migration, and tumor growth in UCEC

**Figure 3:**
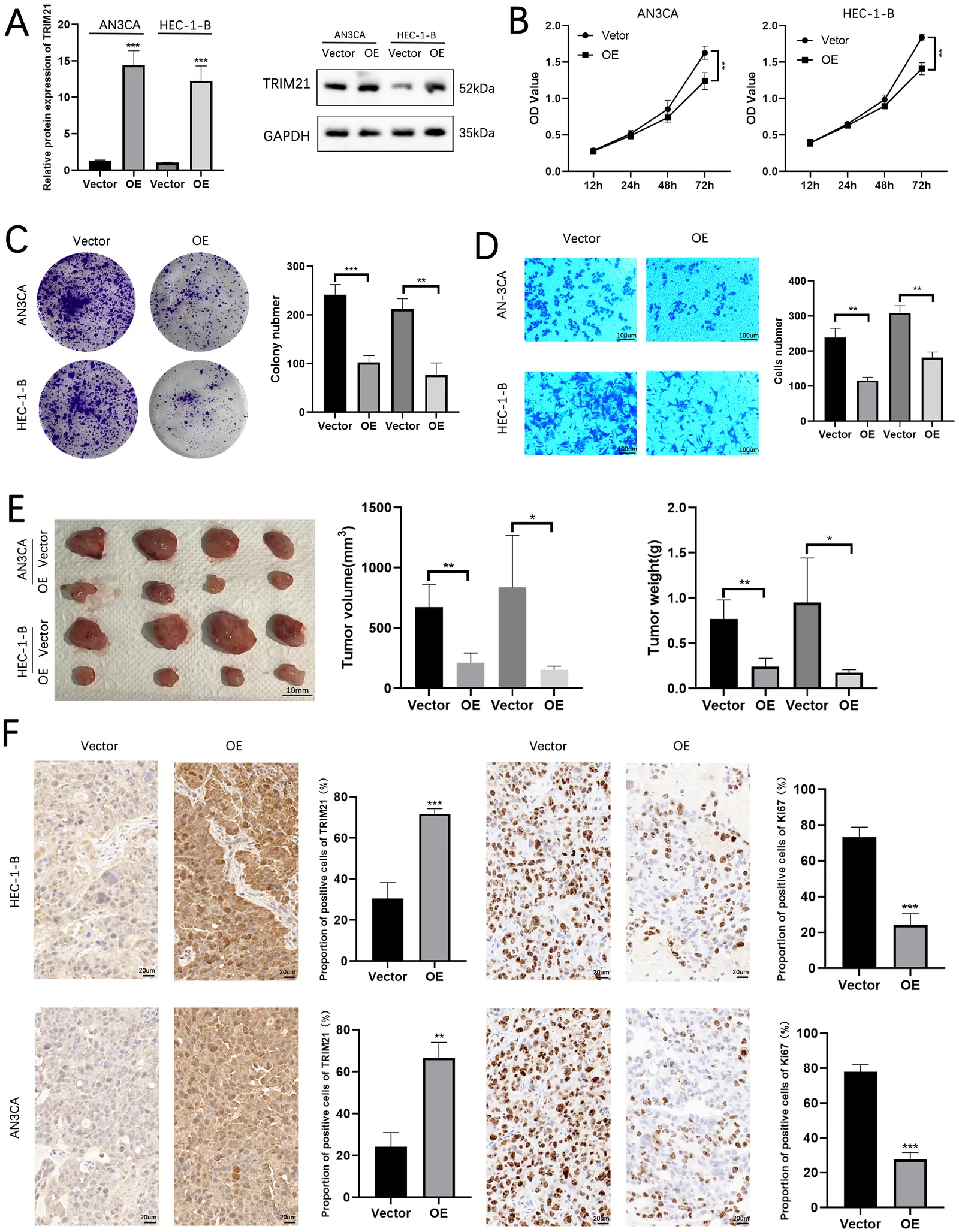
TRIM21 overexpression suppresses malignant phenotypes of UCEC cells *in vitro* and *in vivo*. **(A)** Western blot analysis confirming TRIM21 overexpression in AN3CA and HEC-1-B cells. **(B)** CCK-8 assays showing the effect of TRIM21 overexpression on cell proliferation. **(C)** Representative images and quantitative analysis of colony formation assays in AN3CA and HEC-1-B cells. **(D)** Representative images and quantification of Transwell migration assays in AN3CA and HEC-1-B cells. **(E)** Representative xenograft tumors and quantitative analysis of tumor volume and tumor weight in nude mice injected with control or TRIM21-overexpressing cells. **(F)** Representative immunohistochemical staining images and quantitative analysis of TRIM21 and Ki-67 expression in xenograft tissues. Data are shown as mean ± SD. *P < 0.05, **P < 0.01, ***P < 0.001.

To investigate the biological role of TRIM21 in UCEC, TRIM21 overexpression models were established in AN3CA and HEC-1-B cells. Quantitative real time polymerase chain reaction and Western blot analyses confirmed successful overexpression of TRIM21 in both cell lines (**Figure 3A**). Functional assays showed that TRIM21 overexpression significantly inhibited cell proliferation, as determined by Cell Counting Kit 8 assays (**Figure 3B**). Colony formation assays further demonstrated that TRIM21 overexpression markedly reduced clonogenic capacity (**Figure 3C**). In addition, Transwell assays showed that TRIM21 overexpression significantly suppressed cell migration (**Figure 3D**). To further validate the tumor-suppressive role of TRIM21, loss-of-function experiments were performed. Western blot analysis confirmed efficient TRIM21 knockdown in HEC-1-B and AN3CA cells (**Supplementary Figure 1B**). TRIM21 depletion significantly increased cell proliferation, as indicated by Cell Counting Kit-8 assays (**Supplementary Figure 1C**). Moreover, TRIM21 knockdown enhanced colony formation and migratory capacity in both cell lines (**Supplementary Figures 1D, E**). These findings were consistent with the inhibitory effects observed following TRIM21 overexpression. *In vivo*, xenograft experiments revealed that tumors derived from TRIM21 overexpressing cells exhibited significantly reduced volume and weight compared with those in the control group, indicating that TRIM21 suppresses UCEC tumor growth (**Figure 3E**). Immunohistochemical analysis of xenograft tissues further showed increased TRIM21 positivity and decreased Ki 67 positivity in the TRIM21 overexpression group (**Figure 3F**). These findings indicate that TRIM21 acts as a tumor suppressor in UCEC by inhibiting proliferation and migration.

### TRIM21 promotes apoptosis in UCEC cells

**Figure 4:**
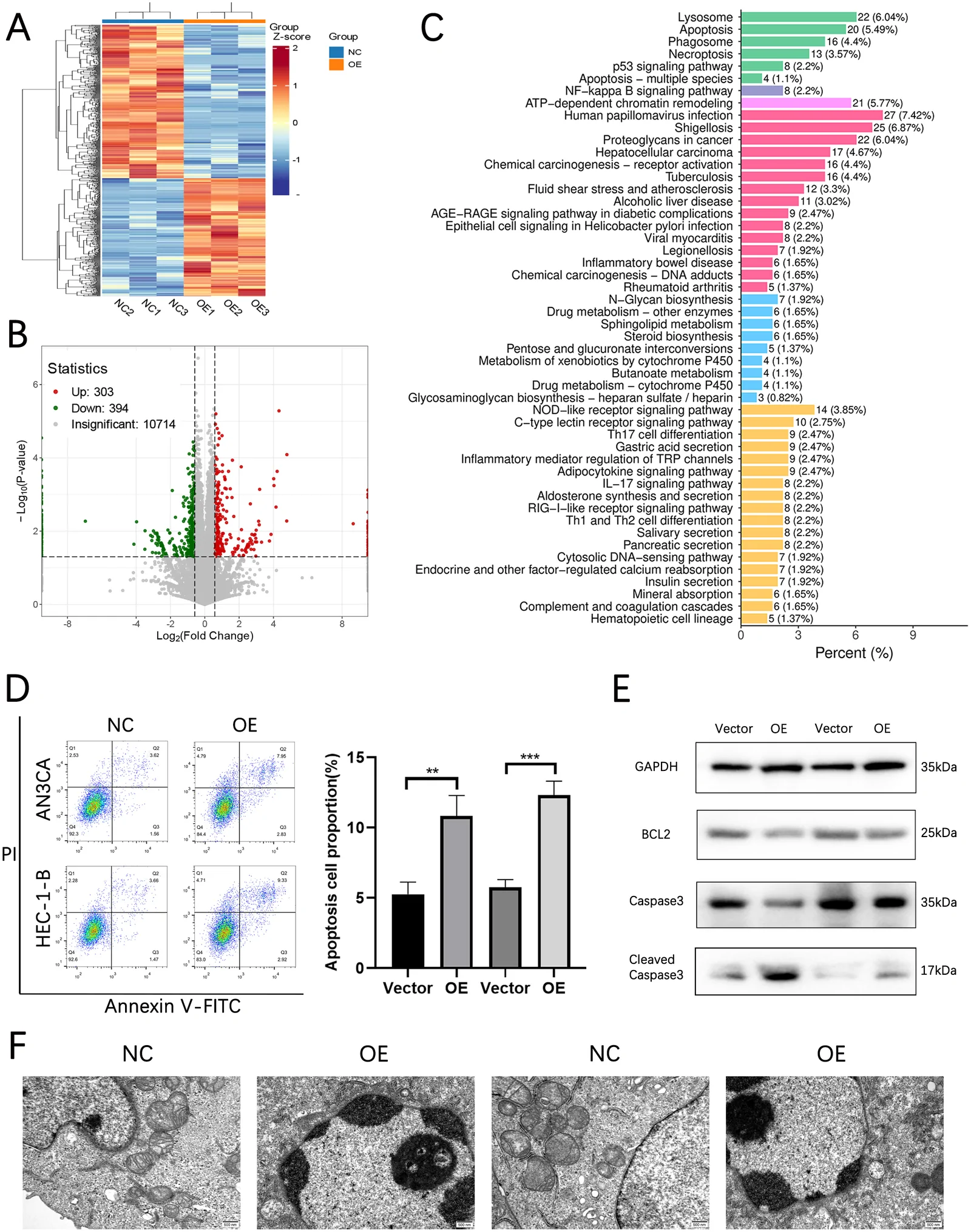
TRIM21 overexpression reshapes the proteomic profile and promotes apoptosis in UCEC cells. **(A)** Heatmap showing differentially expressed proteins in HEC-1-B cells between the TRIM21 overexpression group and the control group. **(B)** Volcano plot of differentially expressed proteins identified by quantitative proteomic analysis. **(C)** KEGG pathway enrichment analysis of differentially expressed proteins. **(D)** Flow cytometric analysis and quantification of apoptosis in AN3CA and HEC-1-B cells after TRIM21 overexpression. **(E)** Western blot analysis of apoptosis-related proteins, including BCL2, Caspase-3, and cleaved Caspase-3, in TRIM21-overexpressing cells. **(F)** Transmission electron microscopy showing ultrastructural apoptotic changes in control and TRIM21-overexpressing UCEC cells. Data are shown as mean ± SD. **P < 0.01, ***P < 0.001.

To explore the molecular basis of TRIM21 mediated tumor suppression, quantitative proteomic analysis was performed in HEC-1-B cells following TRIM21 overexpression. Heatmap analysis revealed substantial alterations in the proteomic profile after TRIM21 overexpression (**Figure 4A**). Volcano plot analysis identified 303 upregulated and 394 downregulated proteins (**Figure 4B**). Kyoto Encyclopedia of Genes and Genomes enrichment analysis showed that these differentially expressed proteins were mainly enriched in apoptosis, lysosome, necroptosis, the p53 signaling pathway, and the nuclear factor kappa B signaling pathway, suggesting that TRIM21 may exert its effects through regulation of cell death related pathways (**Figure 4C**). Flow cytometric analysis further demonstrated that TRIM21 overexpression significantly increased the apoptotic rate of AN3CA and HEC-1-B cells (**Figure 4D**). Western blot analysis showed that TRIM21 overexpression reduced the expression of the antiapoptotic protein BCL2 while increasing the level of cleaved Caspase 3 (**Figure 4E**). Transmission electron microscopy also revealed typical apoptotic ultrastructural changes in TRIM21 overexpressing cells, including chromatin condensation, nuclear shrinkage, and mitochondrial abnormalities (**Figure 4F**). Together, these data indicate that TRIM21 promotes apoptosis in UCEC cells.

### SOAT1 is identified as a interacting partner of TRIM21 in UCEC

**Figure 5:**
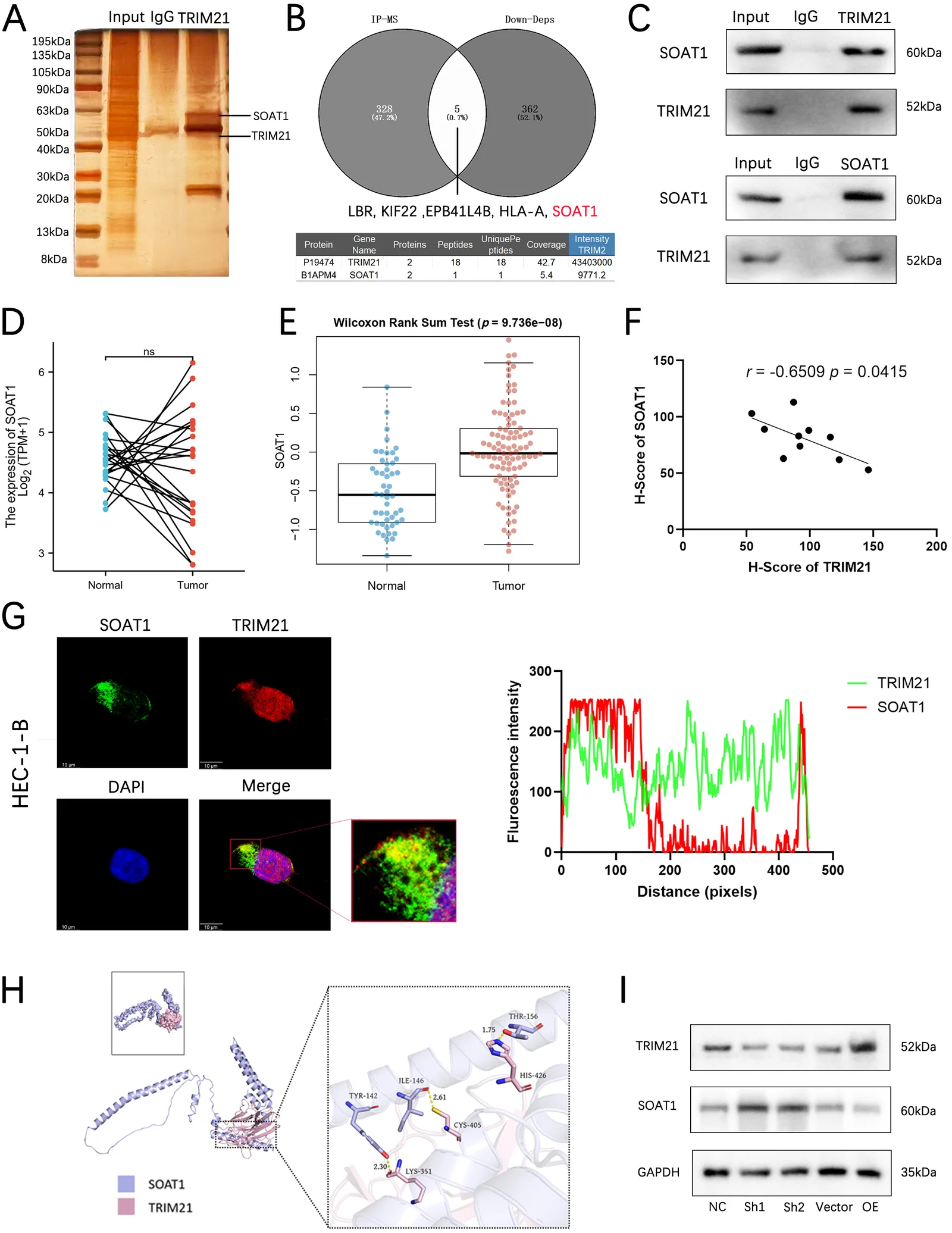
SOAT1 is identified as an interacting protein of TRIM21 in UCEC. **(A)** Silver staining of TRIM21 immunoprecipitates following immunoprecipitation. Differential bands were subjected to mass spectrometry analysis. **(B)** Venn diagram showing the overlap between proteins identified by IP-MS and proteins downregulated after TRIM21 overexpression, highlighting SOAT1 as a candidate downstream target. **(C)** Co-immunoprecipitation assays validating the endogenous interaction between TRIM21 and SOAT1. **(D)** Paired analysis of SOAT1 mRNA expression in TCGA-UCEC samples. **(E)** Validation of SOAT1 protein expression in UCEC using the CPTAC cohort. **(F)** Correlation analysis of TRIM21 and SOAT1 protein expression based on immunohistochemical H-scores. **(G)** Immunofluorescence staining showing the colocalization of TRIM21 and SOAT1 in HEC-1-B cells. Scale bar, 10 μm. **(H)** Molecular docking analysis predicting the binding mode and potential interacting residues between TRIM21 and SOAT1. **(I)** Western blot analysis of SOAT1 expression after TRIM21 knockdown or overexpression. Data are shown as mean ± SD where applicable. *P < 0.05, ***P < 0.001; ns: not significant.

To identify downstream mediators of TRIM21, immunoprecipitation followed by silver staining and mass spectrometry was performed to screen potential interacting proteins. Silver staining revealed multiple specific protein bands in the TRIM21 immunoprecipitates (**Figure 5A**), and mass spectrometry identified several candidate interacting proteins. By intersecting the immunoprecipitation mass spectrometry results with the proteins downregulated upon TRIM21 overexpression, SOAT1 emerged as a candidate downstream target (**Figure 5B**). Co immunoprecipitation assays further confirmed an endogenous interaction between TRIM21 and SOAT1 (**Figure 5C**). At the clinical level, SOAT1 showed no significant difference in paired TCGA UCEC samples (**Figure 5D**), whereas CPTAC data showed significantly elevated SOAT1 protein expression in UCEC tissues (**Figure 5E**). Correlation analysis of immunohistochemical staining revealed a significant negative correlation between TRIM21 and SOAT1 protein expression, with a correlation coefficient of -0.6509 (**Figure 5F**). Immunofluorescence staining further demonstrated clear cytoplasmic colocalization of TRIM21 and SOAT1 (**Figure 5G**), and molecular docking analysis suggested the presence of potential binding sites between these two proteins (**Figure 5H**). Functional regulation experiments showed that TRIM21 knockdown increased SOAT1 protein expression, whereas TRIM21 overexpression reduced SOAT1 protein levels (**Figure 5I**). These results suggest that TRIM21 may regulate UCEC progression by interacting with and negatively regulating SOAT1.

### TRIM21 promotes proteasomal degradation of SOAT1 through K48 linked ubiquitination

**Figure 6:**
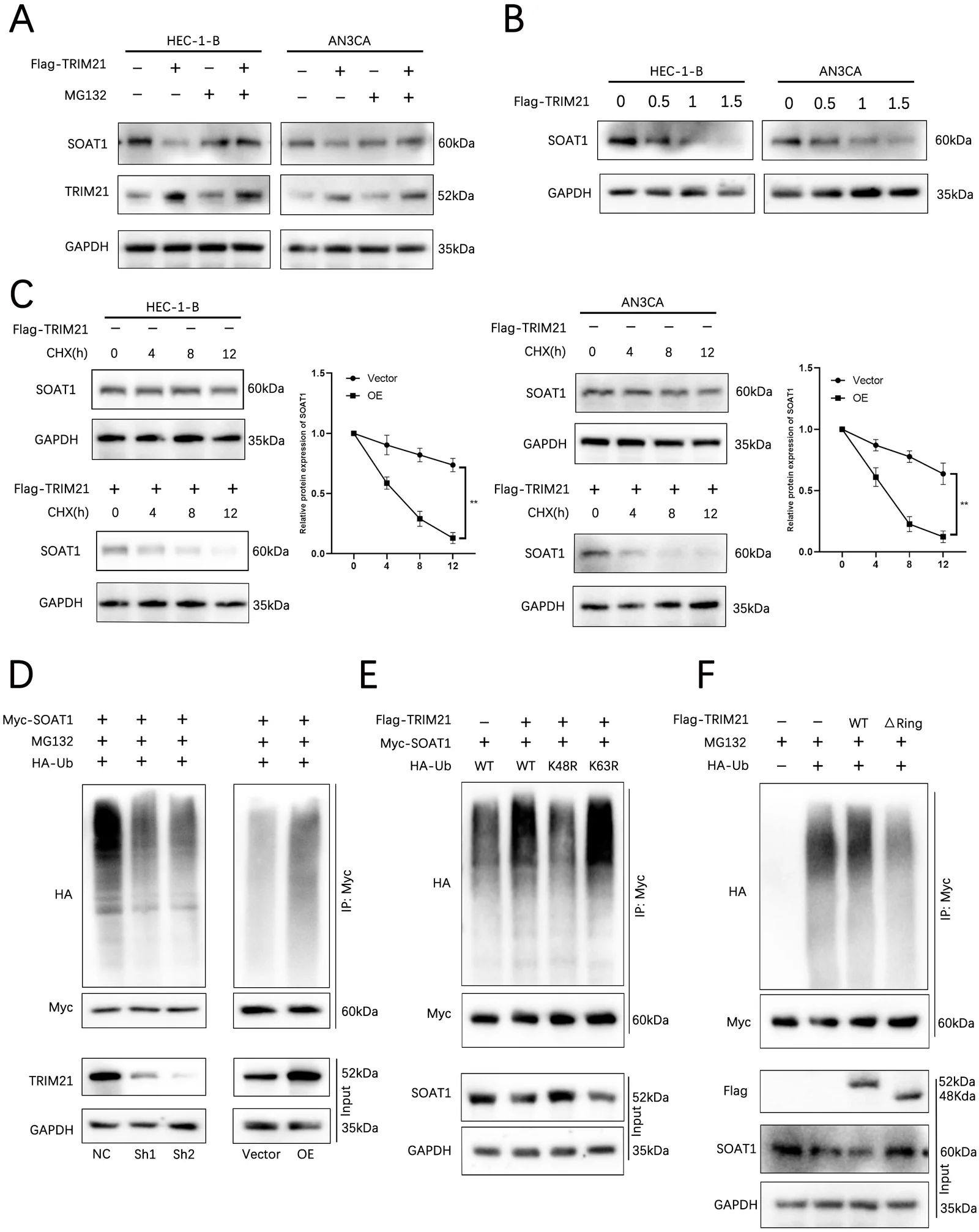
TRIM21 promotes proteasomal degradation of SOAT1 through K48 linked ubiquitination. **(A)** Western blot analysis showing that MG132 treatment reverses the reduction in SOAT1 protein induced by TRIM21 overexpression in HEC-1-B and AN3CA cells. **(B)** Dose-dependent effect of Flag-TRIM21 overexpression on SOAT1 protein levels in HEC-1-B and AN3CA cells. **(C)** Cycloheximide chase assays showing that TRIM21 overexpression accelerates SOAT1 protein degradation and shortens its half-life. **(D)** Ubiquitination assays showing that TRIM21 knockdown reduces, whereas TRIM21 overexpression enhances, SOAT1 ubiquitination. **(E)** Ubiquitin linkage analysis showing that the K48R mutant markedly attenuates TRIM21-mediated SOAT1 ubiquitination and degradation, whereas the K63R mutant has a relatively minor effect. **(F)** Ubiquitination assays showing that deletion of the TRIM21 RING domain attenuates SOAT1 ubiquitination. Data are shown as mean ± SD where applicable. **P < 0.01.

To determine how TRIM21 regulates SOAT1, we first assessed whether the proteasome inhibitor MG132 could block TRIM21 mediated SOAT1 downregulation. Western blot analysis showed that TRIM21 overexpression markedly reduced SOAT1 protein levels in both HEC-1-B and AN3CA cells, whereas MG132 treatment largely reversed this effect, indicating that TRIM21 promotes SOAT1 degradation through a proteasome dependent pathway (**Figure 6A**). We next evaluated whether this regulation was dose dependent. As the amount of Flag tagged TRIM21 plasmid increased, SOAT1 protein levels progressively decreased in both HEC-1-B and AN3CA cells (**Figure 6B**). Cycloheximide chase assays further showed that TRIM21 overexpression accelerated SOAT1 degradation and significantly shortened its protein half life, indicating that TRIM21 reduces SOAT1 protein stability (**Figure 6C**). Given that TRIM21 is an E3 ubiquitin ligase, we next examined whether it modulates SOAT1 ubiquitination. Immunoprecipitation based ubiquitination assays showed that TRIM21 knockdown reduced SOAT1 ubiquitination, whereas TRIM21 overexpression markedly enhanced SOAT1 ubiquitination (**Figure 6D**). To determine the type of ubiquitin linkage involved, different ubiquitin mutants were used. Compared with wild type ubiquitin, the K48R mutant substantially attenuated TRIM21 induced SOAT1 ubiquitination and SOAT1 downregulation, whereas the K63R mutant had a much weaker effect (**Figure 6E**). Moreover, deletion of the RING domain of TRIM21 markedly impaired its ability to induce SOAT1 ubiquitination compared with full-length TRIM21 (**Figure 6F**). These findings demonstrate that the RING domain is required for the E3 ubiquitin ligase activity of TRIM21 toward SOAT1. Collectively, TRIM21 promotes the K48-linked ubiquitination and proteasomal degradation of SOAT1 in a RING domain-dependent manner.

### SOAT1 restoration partially rescues the tumor suppressive effects of TRIM21 in UCEC

**Figure 7:**
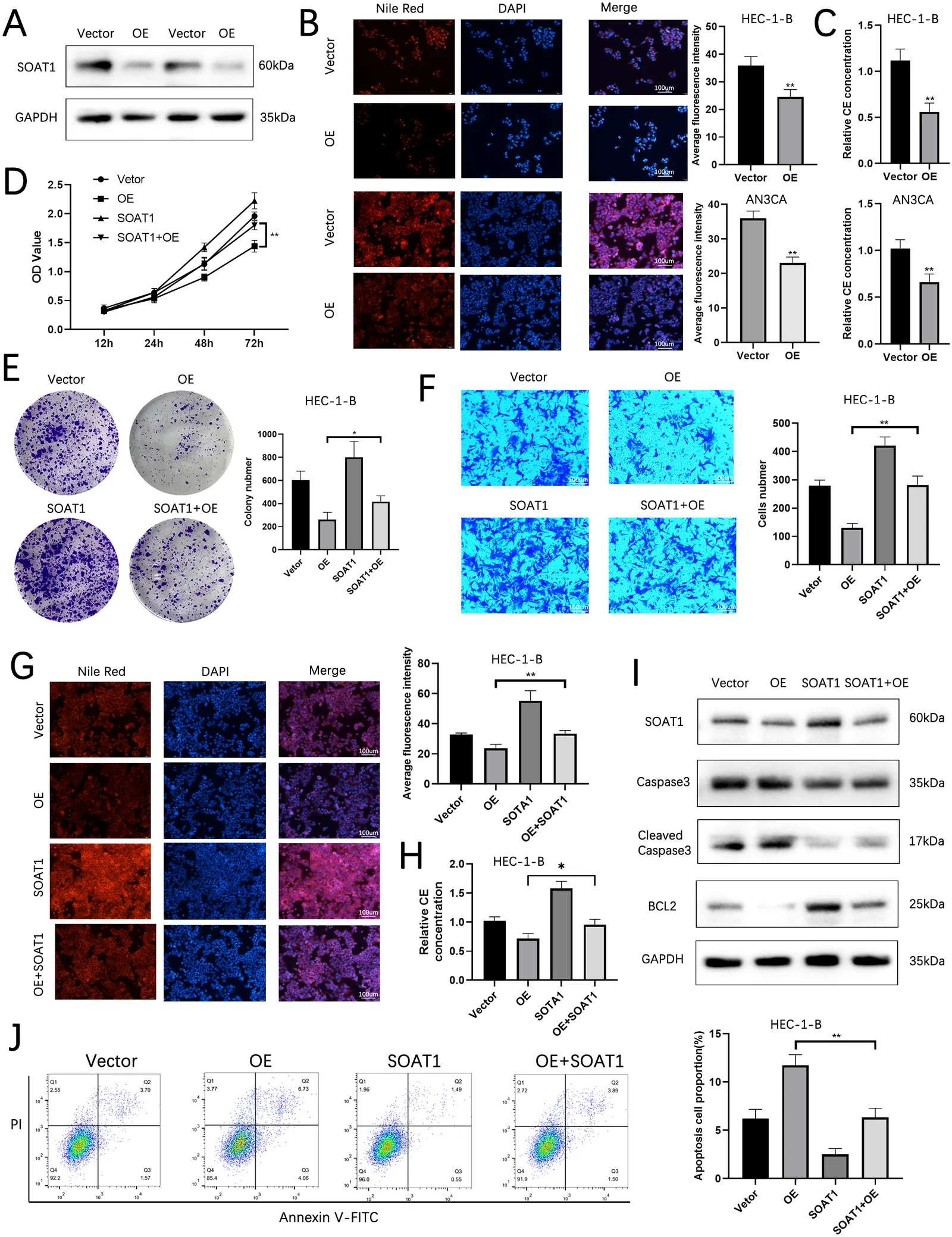
SOAT1 restoration partially rescues the tumor-suppressive effects of TRIM21 in UCEC cells. **(A)** Western blot analysis of SOAT1 after TRIM21 overexpression. **(B)** Nile Red staining and quantification of intracellular lipid accumulation in HEC-1-B and AN3CA cells. **(C)** Intracellular cholesteryl ester (CE) levels after TRIM21 overexpression. **(D)** CCK-8 assays of cell proliferation following SOAT1 restoration. **(E)** Colony formation assays showing partial recovery of clonogenic capacity. **(F)** Transwell assays showing that SOAT1 restoration attenuates TRIM21-mediated inhibition of migration. **(G)** Nile Red staining showing restoration of intracellular lipid accumulation by SOAT1. **(H)** CE levels in the indicated rescue groups. **(I)** Western blot analysis of SOAT1, Caspase-3, cleaved Caspase-3, and BCL2. **(J)** Flow cytometric analysis of apoptosis. Data are presented as mean ± SD. *P < 0.05; **P < 0.01.

To further define the functional significance of SOAT1 in TRIM21-mediated tumor suppression, rescue experiments were performed. Western blot analysis confirmed that TRIM21 overexpression markedly reduced SOAT1 protein levels in HEC-1-B cells, supporting SOAT1 as a downstream effector of TRIM21 (**Figure 7A**). Nile Red staining showed that TRIM21 overexpression significantly decreased intracellular neutral lipid and lipid droplet accumulation in both HEC-1-B and AN3CA cells (**Figure 7B**). Consistently, biochemical analysis demonstrated that TRIM21 overexpression significantly reduced intracellular cholesteryl ester levels in both cell lines (**Figure 7C**), indicating that TRIM21 suppresses SOAT1-dependent cholesterol esterification and lipid storage. We next examined whether restoration of SOAT1 expression could reverse the phenotypic changes induced by TRIM21 overexpression. Cell Counting Kit-8 assays showed that TRIM21 overexpression significantly inhibited HEC-1-B cell proliferation, whereas ectopic SOAT1 expression partially restored proliferative capacity (**Figure 7D**). Similarly, colony formation assays demonstrated that SOAT1 restoration partially reversed the reduction in clonogenic ability caused by TRIM21 overexpression (**Figure 7E**). Transwell assays further showed that the inhibitory effect of TRIM21 overexpression on cell migration was significantly attenuated following SOAT1 restoration (**Figure 7F**). Nile Red staining revealed that SOAT1 overexpression markedly increased intracellular lipid accumulation and partially restored the reduction in lipid storage induced by TRIM21 overexpression (**Figure 7G**). In parallel, measurement of intracellular cholesterol metabolism showed that SOAT1 restoration significantly reversed the decrease in cholesteryl ester content caused by TRIM21 overexpression (**Figure 7H**). These results further indicate that the effects of TRIM21 on intracellular lipid storage are mediated, at least in part, through suppression of SOAT1-dependent cholesterol esterification. Western blot analysis showed that TRIM21 overexpression increased cleaved Caspase-3 expression and decreased BCL2 expression, whereas SOAT1 restoration partially reversed these apoptosis-related protein changes (**Figure 7I**). Consistently, flow cytometric analysis demonstrated that TRIM21 overexpression significantly increased the apoptotic rate of HEC-1-B cells, while SOAT1 overexpression attenuated this pro-apoptotic effect (**Figure 7J**). Collectively, these findings indicate that SOAT1 restoration partially rescues the effects of TRIM21 overexpression on cholesterol esterification, intracellular lipid storage, proliferation, migration, and apoptosis, supporting SOAT1 as an important functional mediator of TRIM21-mediated tumor suppression in UCEC.

### TRIM21 expression in UCEC cells suppresses M2-like macrophage polarization

**Figure 8:**
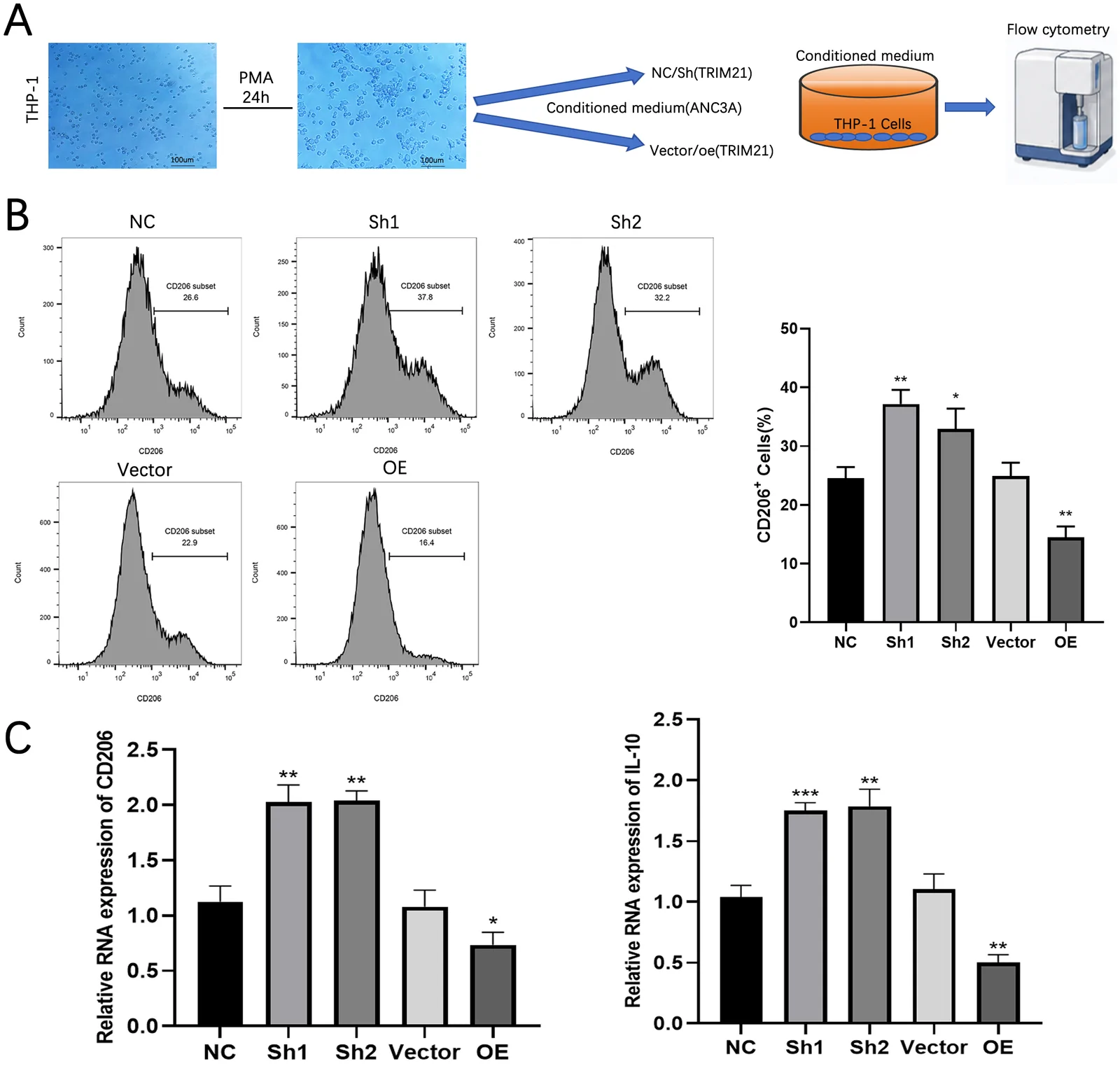
TRIM21 expression in UCEC cells suppresses M2-like macrophage polarization. **(A)** Schematic of the conditioned-medium experiment using PMA-differentiated THP-1 cells. **(B)** Flow cytometric analysis and quantification of CD206-positive macrophages after treatment with conditioned media from AN3CA cells with TRIM21 knockdown or overexpression. **(C)** Quantitative real-time PCR analysis of the M2-associated markers CD206 and IL-10. Data are presented as mean ± SD. *P < 0.05; **: *P* < 0.01; ***P < 0.001.

Previous studies have indicated that SOAT1 participates in the regulation of tumor-associated macrophage (TAM) polarization (34, 35). Given that TRIM21 promotes the ubiquitination and degradation of SOAT1, we further investigated whether tumor-cell TRIM21 could influence macrophage polarization. Conditioned media were collected from AN3CA cells with TRIM21 knockdown or overexpression and subsequently applied to PMA-differentiated THP-1-derived macrophages (**Figure 8A**). Flow cytometric analysis showed that conditioned media from TRIM21-knockdown cells significantly increased the proportion of CD206-positive macrophages compared with the negative control, whereas conditioned media from TRIM21-overexpressing cells markedly reduced the CD206-positive population compared with the vector control (**Figure 8B**). Consistently, quantitative real-time PCR analysis demonstrated that conditioned media from TRIM21-knockdown cells increased the expression of the M2 macrophage markers CD206 and IL-10. In contrast, conditioned media from TRIM21-overexpressing cells significantly decreased CD206 and IL-10 expression (**Figure 8C**). Collectively, these findings suggest that loss of TRIM21 in UCEC cells promotes M2-like macrophage polarization through tumor cell-derived soluble factors, whereas TRIM21 overexpression suppresses this M2-like macrophage phenotype.

## Discussion

UCEC progression is driven by a complex network of molecular events involving dysregulated gene expression, altered protein homeostasis, metabolic reprogramming, and changes in the tumor microenvironment (36, 37). Although molecular classification has greatly improved the understanding of UCEC heterogeneity, the regulatory mechanisms that connect protein degradation with metabolic abnormalities remain insufficiently defined. The present study supports a tumor suppressive role of TRIM21 in UCEC and suggests that its biological significance extends beyond reduced expression alone. Gain- and loss-of-function experiments consistently demonstrated that TRIM21 inhibits UCEC cell proliferation, clonogenic growth, and migration, while *in vivo* experiments further confirmed its inhibitory effect on tumor growth. Moreover, multivariate regression analysis identified TRIM21 as an independent prognostic factor in patients with UCEC. The major implication lies in the finding that TRIM21 regulates the stability of a lipid metabolism-related protein and thereby influences proliferation, migration, apoptosis, cholesterol esterification, and tumor–macrophage communication.

TRIM21 is a functionally important E3 ubiquitin ligase whose role in cancer is strongly dependent on tumor type and molecular context. Previous studies have shown that TRIM21 can suppress malignant progression by promoting the degradation of oncogenic substrates such as mutant p53, PRMT1, and SIX2 (21–23). In addition, TRIM21 has been implicated in the regulation of tumor metastasis, stemness, immune response, and treatment sensitivity through substrate-specific ubiquitination. However, opposite effects have also been reported in other tumor settings, where TRIM21 facilitates tumor adaptation by modulating proteins involved in metabolism or resistance to cell death. This functional diversity indicates that TRIM21 cannot be simply categorized as either oncogenic or tumor suppressive. Instead, its biological role must be interpreted according to the identity of its substrates and the signaling environment of specific cancers. In UCEC, the present findings are more consistent with a tumor suppressive function. Reduced TRIM21 expression may weaken the degradation of pro-tumorigenic proteins, thereby favoring sustained growth and survival of tumor cells.

Abnormal lipid metabolism is increasingly recognized as a major contributor to cancer progression (38, 39). Compared with glucose and amino acid metabolism, lipid metabolism not only supplies membrane components and energy reserves but also regulates membrane dynamics, redox balance, and stress adaptation (40, 41). Cholesterol metabolism is particularly important in this context. SOAT1 converts free cholesterol into cholesteryl esters and facilitates lipid droplet formation, thereby reducing lipotoxicity and enhancing cellular tolerance to metabolic stress (42). Increased SOAT1 expression has been associated with enhanced proliferation, migration, treatment resistance, and poor prognosis in several malignancies (30, 31, 43). The present findings indicate that TRIM21 and SOAT1 are functionally linked in UCEC, suggesting that dysregulated protein degradation and abnormal cholesterol metabolism may cooperate in maintaining the malignant phenotype.

Mechanistically, TRIM21 was found to promote K48 linked ubiquitination of SOAT1 and to induce its proteasomal degradation. K48 linked ubiquitin chains represent the canonical signal for proteasomal recognition and substrate degradation. This indicates that TRIM21 regulates SOAT1 through a classical degradation-directed ubiquitination mechanism rather than through a non-degradative modification. At the same time, TRIM21 is not restricted to K48 linked ubiquitination. Earlier studies have shown that TRIM21 can also mediate K63 linked or other non-canonical ubiquitin modifications to regulate substrate activation, localization, or signaling output (44, 45). Therefore, the identification of SOAT1 as a K48 linked ubiquitination substrate in UCEC further supports the substrate selective and linkage selective nature of TRIM21.

The biological significance of SOAT1 regulation by TRIM21 is not limited to cholesterol metabolism itself. Cholesterol homeostasis and lipid droplet dynamics are closely related to mitochondrial function, membrane composition, oxidative stress, and apoptosis sensitivity (46, 47). High SOAT1 activity facilitates the conversion of free cholesterol into cholesteryl esters, thereby buffering lipotoxic stress and improving the capacity of tumor cells to adapt to unfavorable conditions. The observed decrease in lipid droplet accumulation together with the increase in apoptosis following TRIM21-mediated SOAT1 degradation suggests that TRIM21 may impair metabolic adaptation and render tumor cells more susceptible to apoptotic stimuli. This interpretation provides a plausible explanation for the simultaneous effects of TRIM21 on proliferation, migration, and apoptosis. In this regard, TRIM21 appears to act not only as a regulator of protein degradation but also as an upstream modulator of lipid metabolism and cell fate determination in UCEC.

The role of TRIM21 may also extend beyond tumor cell-intrinsic regulation. Previous studies have implicated TRIM21 in inflammation and antitumor immunity (17, 48). In certain tumor models, TRIM21 has been shown to influence immune signaling pathways and immune cell activation by regulating the stability of key proteins (49, 50). SOAT1 has also been implicated in the regulation of tumor-associated macrophage polarization, suggesting that cholesterol esterification may influence not only tumor-cell metabolism but also the immune microenvironment. In the present study, conditioned media from TRIM21-knockdown UCEC cells increased the proportion of CD206-positive THP-1-derived macrophages and elevated the expression of the M2-associated markers CD206 and IL-10. Conversely, conditioned media from TRIM21-overexpressing cells suppressed these M2-like polarization features. These results suggest that tumor-cell TRIM21 can alter secreted signals that regulate macrophage phenotype and that reduced TRIM21 expression may contribute to the formation of an immunosuppressive microenvironment. The single-cell and stemness-related analyses in the current study suggest that TRIM21 displays heterogeneous expression among different cell populations and may be associated with selected immune cell subsets and epigenetic stemness features. Nevertheless, because SOAT1-specific rescue or inhibition was not performed in the conditioned-medium model, the current data do not yet establish that the effect of TRIM21 on macrophage polarization is directly mediated by SOAT1.

From a translational perspective, both TRIM21 and SOAT1 may have clinical relevance. Reduced TRIM21 expression and its association with adverse prognosis and greater invasiveness suggest that TRIM21 may serve as an auxiliary biomarker reflecting tumor aggressiveness. SOAT1, as a metabolic enzyme, may be more directly amenable to pharmacological intervention. When TRIM21 expression is low and SOAT1 stability is consequently increased, direct targeting of SOAT1 may represent a rational therapeutic strategy. In addition, TRIM21 itself has attracted attention as a potentially druggable E3 ubiquitin ligase, and TRIM21-related pathways have already been explored in the context of targeted protein degradation strategies (51, 52). These considerations indicate that further investigation of TRIM21 and SOAT1 may contribute to the development of precision therapy for UCEC.

Several limitations should be acknowledged. Larger independent cohorts are needed to validate the prognostic significance of TRIM21 and SOAT1. Although lipid droplets, cholesteryl ester levels, and SOAT1 rescue were examined, broader lipidomic and metabolomic analyses are required. Additional TRIM21 substrates may also contribute to UCEC progression. Moreover, the soluble mediators linking tumor-cell TRIM21 to M2-like macrophage polarization, and the direct involvement of SOAT1, remain to be clarified in primary macrophages, co-culture systems, immune-competent models, and rescue experiments.

In summary, TRIM21 functions as a tumor suppressor in UCEC by promoting RING domain-dependent K48-linked ubiquitination and proteasomal degradation of SOAT1. This mechanism suppresses cholesterol esterification-related metabolic adaptation, inhibits proliferation and migration, promotes apoptosis, and reduces M2-like macrophage polarization. These findings highlight the TRIM21–SOAT1 axis as a link between protein homeostasis, lipid metabolism, and the tumor immune microenvironment in UCEC.

## Data Availability

The original contributions presented in the study are included in the article/**Supplementary Material**. Further inquiries can be directed to the corresponding author.

## Supplementary material

Supplementary Table 1 Complete quantitative proteomic expression matrix of TRIM21-OE and control HEC-1-B cells.

Supplementary Table 2 Differentially expressed proteins between TRIM21-OE and control HEC-1-B cells.

Supplementary Table 3 Candidate TRIM21-interacting proteins identified by immunoprecipitation–mass spectrometry.

Supplementary Figure 1 TRIM21 expression is independently associated with UCEC prognosis, and TRIM21 knockdown promotes malignant phenotypes in UCEC cells. **(A)** Forest plot of multivariable Cox regression analysis evaluating the independent prognostic value of TRIM21 expression in patients with UCEC. **(B)** Western blot analysis and quantification confirming TRIM21 knockdown in HEC-1-B and AN3CA cells. **(C)** CCK-8 assays showing increased cell proliferation after TRIM21 knockdown. **(D)** Representative images and quantification of colony formation assays showing enhanced clonogenic capacity following TRIM21 depletion. **(E)** Representative images and quantitative analysis of Transwell assays showing increased cell migration after TRIM21 knockdown. Data are presented as mean ± SD. **P* < 0.05; ***P* < 0.01.
